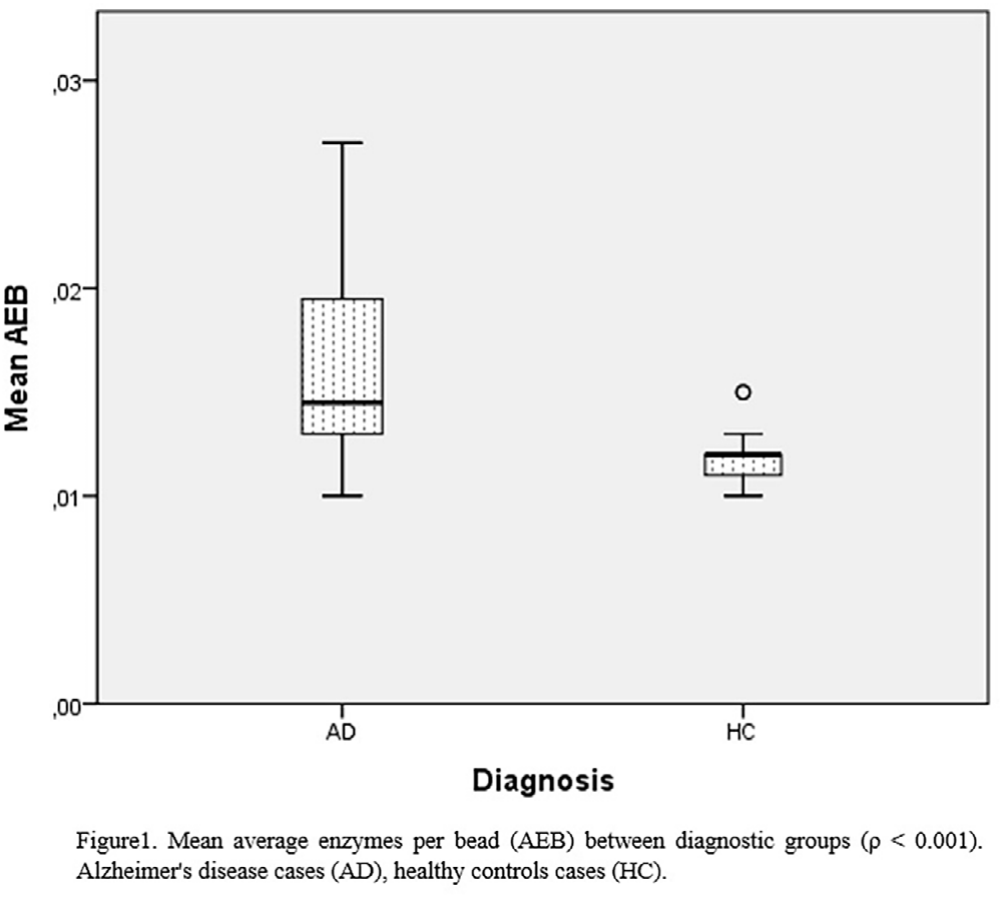# D13 caspase‐6‐cleaved tau, a new biomarker to complement Alzheimer’s disease diagnosis

**DOI:** 10.1002/alz.095421

**Published:** 2025-01-09

**Authors:** Liara Rizzi, Julio C. Rojas, Andrew J. Ambrose, Marcio Luiz Figueredo Balthazar, Michelle R. Arkin, Lea T. Grinberg

**Affiliations:** ^1^ Memory and Aging Center, UCSF Weill Institute for Neurosciences, University of California, San Francisco, San Francisco, CA USA; ^2^ University of Campinas (Unicamp), Campinas, SP Brazil; ^3^ UCSF Department of Pharmaceutical Chemistry and Small Molecule Discovery Center, University of California, San Francisco, San Francisco, CA USA; ^4^ Physiopathology in Aging Laboratory (LIM‐22), University of São Paulo Medical School, São Paulo, São Paulo Brazil; ^5^ Department of Pathology, University of California San Francisco, San Francisco, CA USA

## Abstract

**Background:**

Caspase‐cleaved tau can critically be involved in the pathogenesis and progression of Alzheimer’s disease (AD), due to its ability to promote both misfolding and neurodegeneration, which ultimately leads to progressive cognitive impairment. Neuropathological studies show that caspase‐cleaved tau species are abundant in AD brain neurons, yet show only a modest degree of co‐occurrence with phospho‐tau. This suggests that caspase‐cleaved tau is an overlooked form of tau pathology that is related to the vulnerability and pathogenesis of AD.

**Method:**

We developed a homebrew Simoa immunoassay for detecting N‐terminal D13 caspase‐6‐cleaved tau in the cerebrospinal fluid (CSF). For developing the assay, we used a neo‐epitope monoclonal antibody developed and validated by our group and used in previously published histological work. After optimization, we tested this assay in a pilot study with a discovery cohort comprising CSF samples from 20 subjects with mild to moderate dementia and positive for AD biomarkers and 20 well‐characterized healthy controls (HC).

**Result:**

The signal translated in the mean average enzymes per bead was significantly higher in Alzheimer’s disease cases compared to healthy controls (0.01640 vs. 0.01189, ρ < 0.001). There were no significant differences in demographics such as age (ρ = 0.072) or gender (ρ = 0.723) between the two groups.

**Conclusion:**

We introduce the initial D13 caspase‐6‐cleaved tau biomarker immunoassay for quantitatively assessing caspase‐cleaved tau pathology in CSF. This assay offers the chance to identify pathological tau that conventional p‐tau assays may overlook, potentially enhancing the assessment of clinical trials. We are currently expanding the study cohort and correlating the findings with metrics from p‐tau biomarkers.